# Heat Acclimation-Mediated Cross-Tolerance: Origins in within-Life Epigenetics?

**DOI:** 10.3389/fphys.2017.00548

**Published:** 2017-07-28

**Authors:** Michal Horowitz

**Affiliations:** Laboratory of Environmental Physiology, Faculty of Dentistry, Hebrew University of Jerusalem Jerusalem, Israel

**Keywords:** heat acclimation, heat acclimation-mediated cross-tolerance, epigenetic mechanisms of gene expression, HSP72, HIF-1α, attenuated Ca^2+^ overload injuries

## Abstract

The primary outcome of heat acclimation is increased thermotolerance, which stems from enhancement of innate cytoprotective pathways. These pathways produce “ON CALL” molecules that can combat stressors to which the body has never been exposed, via cross-tolerance mechanisms (heat acclimation-mediated cross-tolerance—HACT). The foundation of HACT lies in the sharing of generic stress signaling, combined with tissue/organ- specific protective responses. HACT becomes apparent when acclimatory homeostasis is achieved, lasts for several weeks, and has a memory. HACT differs from other forms of temporal protective mechanisms activated by exposure to lower “doses” of the stressor, which induce adaptation to higher “doses” of the same/different stressor; e.g., preconditioning, hormesis. These terms have been adopted by biochemists, toxicologists, and physiologists to describe the rapid cellular strategies ensuring homeostasis. HACT employs two major protective avenues: constitutive injury attenuation and abrupt post-insult release of help signals enhanced by acclimation. To date, the injury-attenuating features seen in all organs studied include fast-responding, enlarged cytoprotective reserves with HSPs, anti-oxidative, anti-apoptotic molecules, and HIF-1α nuclear and mitochondrial target gene products. Using cardiac ischemia and brain hypoxia models as a guide to the broader framework of phenotypic plasticity, HACT is enabled by a metabolic shift induced by HIF-1α and there are less injuries caused by Ca^+2^ overload, via channel or complex-protein remodeling, or decreased channel abundance. Epigenetic markers such as post-translational histone modification and altered levels of chromatin modifiers during acclimation and its decline suggest that dynamic epigenetic mechanisms controlling gene expression induce HACT and acclimation memory, to enable the rapid return of the protected phenotype. In this review the link between *in vivo* physiological evidence and the associated cellular and molecular mechanisms leading to HACT and its difference from short-acting cross-tolerance strategies will be discussed.

## Introduction

Research into the important topic of acclimation/acclimatization to an adverse environment began in the late Nineteenth century, in the wake of colonialization, and the necessity to adapt to harsh occupational environments in hot tropical countries. The concept was the subject of medical and scientific debates. Based on the experimental physiological evidence existing at the time, the interactions between stressor(s) and the corresponding integrative response(s) were termed, “cross-adaptation” and/or “cross-tolerance” (Adolpf, 1964; Fregly, 1996), even though the exposures occurred over varying lengths of time, and no differentiation was made between simultaneous or sequential exposures to the co-adaptagents.

**Figure 1:**
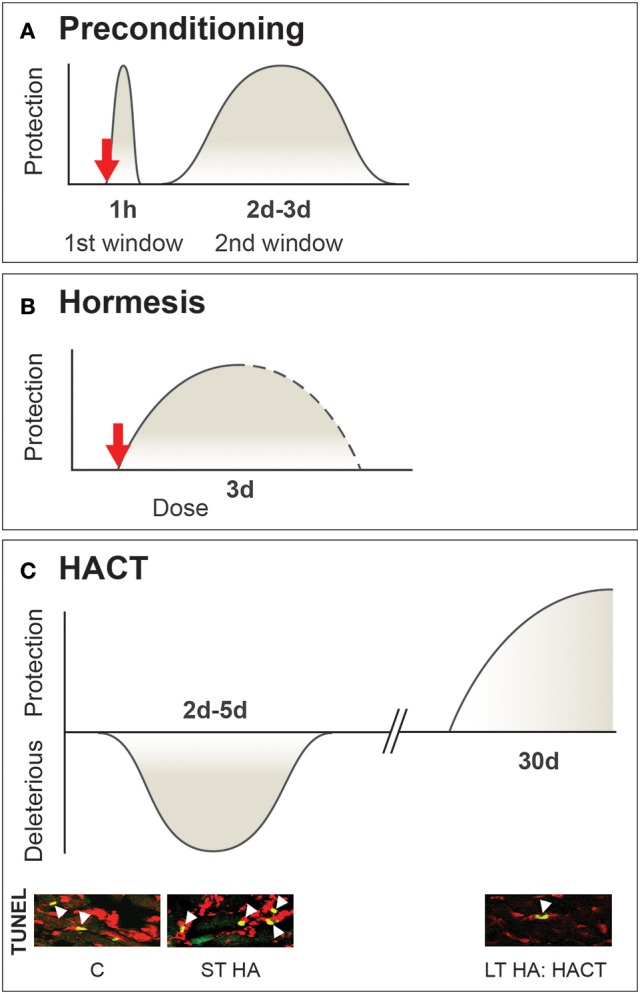
Multifaceted timeframe and mechanisms of β adrenergic signaling cross-tolerance While preconditioning and hormesis are transient short acting cross-tolerance strategies activated upon perturbations to defense cellular homeostasis, HACT takes place only when acclimatory homeostasis has been achieved. **(A)** Preconditioning, with sub-lethal stress induces two windows of protection. The first involves adenosine receptors, K_ATP_ mitochondrial channels, and salvage kinases. The second (delayed) window of protection is associated with transcriptional and translational processes involving cytoprotective molecules. **(B)** Hormesis a dose-dependent stress response initiated upon stress exposure (i.e., toxins only). **(C)** HACT, established following the bi-phasic HA acclimation process, initially signified by enhanced neural activity and triggered molecular processes to maintain DNA integrity. These processes are replaced by the enhancement of innate cytoprotective features. HA temperature is the upper point of the temperature neutral zone (TNZ) of the species. Based on (Horowitz, 1998; Yellon and Downey, 2003; Bolli, 2007; Calabrese et al., 2007).

This lack of clarity in definition enabled multiple interpretations of the relevant terms. For example, some authors use “cross-acclimation” when physiological strain is attenuated (Lee et al., 2016) while others employ “cross-tolerance” when improved cellular protection is observed (Selye and Bajusz, 1961; Ely et al., 2014). Another phenomenon included within the concept of cross-tolerance, formerly termed “cross-resistance” [originally defined by Selye in 1961 (Selye and Bajusz, 1961)], is now known as “preconditioning” (Murry et al., 1986) or “hormesis”, entailing adaptation to a high, even lethal dose of a stressor, following exposure to a lower dose of that stressor (Calabrese, 2016). To further complicate matters, physiologists use “preconditioning”, while its synonym, “hormesis,” was adopted by biochemists and toxicologists. Both describe *a rapidly evoked* cellular strategy that ensures homeostasis, in contrast to acclimation-induced cross-tolerance, which, takes place after acclimation homeostasis has been achieved (Figure 1).

Advances in our understanding of cellular, molecular and epigenetic mechanisms over the last century, have enabled researchers to fine-tune the meanings of these terms. Accordingly, this mini-review aims to clarify the differences between acclimation and environmentally induced cross-tolerance and other modes of cross-tolerance, using as models heat acclimation-mediated cross-tolerance in rodents and in humans. To do so, the link between *in vivo* physiological evidence and the associated cellular and molecular mechanisms will be discussed, initially regarding specific stress-protected targets, and then within the broader framework of acclimatory phenotype plasticity.

## Heat acclimation-mediated cross-tolerance vs. preconditioning-induced cross-tolerance

Heat acclimation (HA) is a reversible, within-lifetime phenotypic adaptation to high ambient temperatures. The primary physiological signature of HA is increased heat endurance via prolongation of the operating thermoregulatory span of physiological mechanisms, combined with improved thermotolerance associated with enhanced molecular cytoprotective mechanisms (Horowitz, 2014, 2016). An important feature of the HA phenotype is the acquired cytoprotection to novel stressors (i.e., stressors to which the body has never been exposed) via cross-tolerance mechanisms (so-called heat acclimation-mediated cross-tolerance, or HACT). This cytoprotection stems from the enhancement of innate cytoprotective pathways that also confer cellular thermotolerance. Based on a sharing principle, these cytoprotective pathways produce “on call” molecules to counteract the consequences of other forms of stress. Gene chip experiments and bioinformatic analyses have collectively demonstrated that HACT is conferred by sharing generic signaling pathways involved in the stress response, with tissue-specific protective pathways (Horowitz et al., 2004). Evidence from animals and humans confirms its effectivity in cardio- and neuroprotection under conditions of hypoxic, hyperoxic, ischemic and traumatic brain injury (TBI; Horowitz, 2014).

HACT (as well as acclimation-induced cross-tolerance in general) differs from the cross-tolerance that occurs following classical preconditioning [analogous to the heat shock response (HSR)], a two-window transient effect (Figure 1A) evoked by short exposures to sublethal stress, thus protecting the subject from an otherwise fatal exposure to a second stress, either similar or different in nature from the original conditioning stress (Bolli, 2007). The first window of protection, involving salvage kinases and adenosine receptors, is rapid, and lasts for ~1 h. The second window of protection appears after 24 h, lasts ~48 h, and relies on transcriptional activation of cytoprotective pathways (Das and Das, 2008).

In contrast, HACT develops slowly during exposure to the acclimating stressors, and is shaped by the nature of the acclimation, as it occurs. HA is a biphasic process, with an initial, transient phase, during which intensive neural stimulation controls physiological processes to alleviate increased body temperature, while molecular pathways are recruited to maintain DNA integrity. This phase, known as “short-term HA,” takes place over ~5 days, and constitutes the “on” switch, which triggers the lengthier processes necessary to achieve acclimatory homeostasis (~3–4 weeks in sedentary rats; Horowitz, 2014). HACT only becomes apparent after the longer second phase, as demonstrated in rodents by Assayag et al. (2010) in their discussion of HA-mediated cardioprotection in cardiac mitochondria, and in the frontal cortex and hippocampus, shown by Yacobi et al. (2014) in studies of HA-mediated neuroprotection. Acclimation in humans displays short and long phases, as well, e.g., Wyndham et al. (1976); however, HA protocols in humans include exercise training last ~2 weeks and no data are as yet available to assess whether the cellular acclimatory homeostasis stage has been completely achieved following that period of time.

Exceptional features of HACT are that it lasts for ~2–3 weeks, depending on the specific feature studied after the HA session (Cohen, 2002), and is memorized. In the rat HA-reacclimation model, 2 days of re-acclimation subsequent to 1 month of HA, and 1 or 2 months of exposure to normothermic ambient temperatures, are sufficient to restore a protected phenotype (Tetievsky et al., 2008). The transient phase of short-term acclimation which, via autonomic control, compensates for thermoregulatory effector-cellular perturbations, only aggravates the response to novel cellular stress, as exemplified in Figure 1C by the enhanced apoptosis in ischemic/reperfused or heat-stressed hearts, on short term HA. However, it doesn't abolish but enhances physiological thermoregulatory responses; i.e., the evaporative cooling or peripheral vasodilation controlled by the central autonomic thermoregulatory centers. This short-term HA impact was confirmed for both passive acclimation (e.g., in rats, which are capable of enhancing evaporation and enduring heat stress longer than rats undergoing long-term HA (Horowitz et al., 1983) and active acclimation (namely, HA and exercise training), described in Garrett et al. for humans enhancing their exercise capacity due to improved cardiovascular performance, following short-term heat acclimation (Garrett et al., 2012).

To date, accumulating experimental evidence and bioinformatic analyses of the HA transcriptome have established that, irrespective of the organ studied, HACT employs a two-tier protective response: (i) constitutive injury attenuation; and (ii) the abrupt, post-insult release of help signals that are enhanced in the acclimated phenotype. Moreover, recent experimental evidence regarding “injury attenuation” (Kodesh et al., 2011; Yacobi et al., 2014) demonstrates the involvement of groups of mechanisms that operate according to similar principles.

## HACT: constitutive injury attenuation—a lesson from the ischemic heart

### Cytoprotection and heat shock proteins (HSP)

Constitutive enhancement of cytoprotective networks enables recruitment of help signals to maintain cellular homeostasis, without the need for *de novo* induction of cytoprotective elements. Chronologically, the first publications regarding acclimatory plasticity leading to constitutive cytoprotection were a series of studies by Maloyan et al. (Maloyan et al., 1999; Maloyan and Horowitz, 2002), demonstrating profound augmentation of HSP72 reserves in rat hearts, following long-term heat acclimation. Sustained low-plasma thyroxine levels, characteristic of acclimatory homeostasis, play a pivotal role in alterations in the density and affinity of the adrenergic receptors and, in turn, lead to altered responsiveness of the HA phenotype to sympathetic signaling, which includes HSP72 induction as well (Maloyan and Horowitz, 2002). Collectively, in the HA phenotype, sustained low thyroxine levels diminish thyroxine's effects on the β-adrenergic pathway, leading to depressed *hsp72* transcription (Maloyan and Horowitz, 2002). Activation of this branch of the thyroxine pathway (namely down regulation of HSP72 transcription, in contrast to enhancement of β adrenergic induced HSP72 transcription at high thyroxine levels) constitutes one explanation for the abolishment of cardioprotection, when HA is conducted with a β adrenergic blockade. This pharmacological tool provides unequivocal proof of the importance of HSP70s to the HACT response against ischemia. However, accumulating evidence implies that anti-oxidative and anti-apoptotic pathways associated with HSP70s are part of their functional performance via downstream pathways—e.g., anti-apoptosis—that inhibit the activation of caspase-9 or caspase-3 by preventing apoptosome formation or cytochrome C release through binding to the apoptosome (Saleh et al., 2000), mitochondrial protein importation (Schulz et al., 2015) or anti-oxidation (Chong et al., 2013).

**Figure 2:**
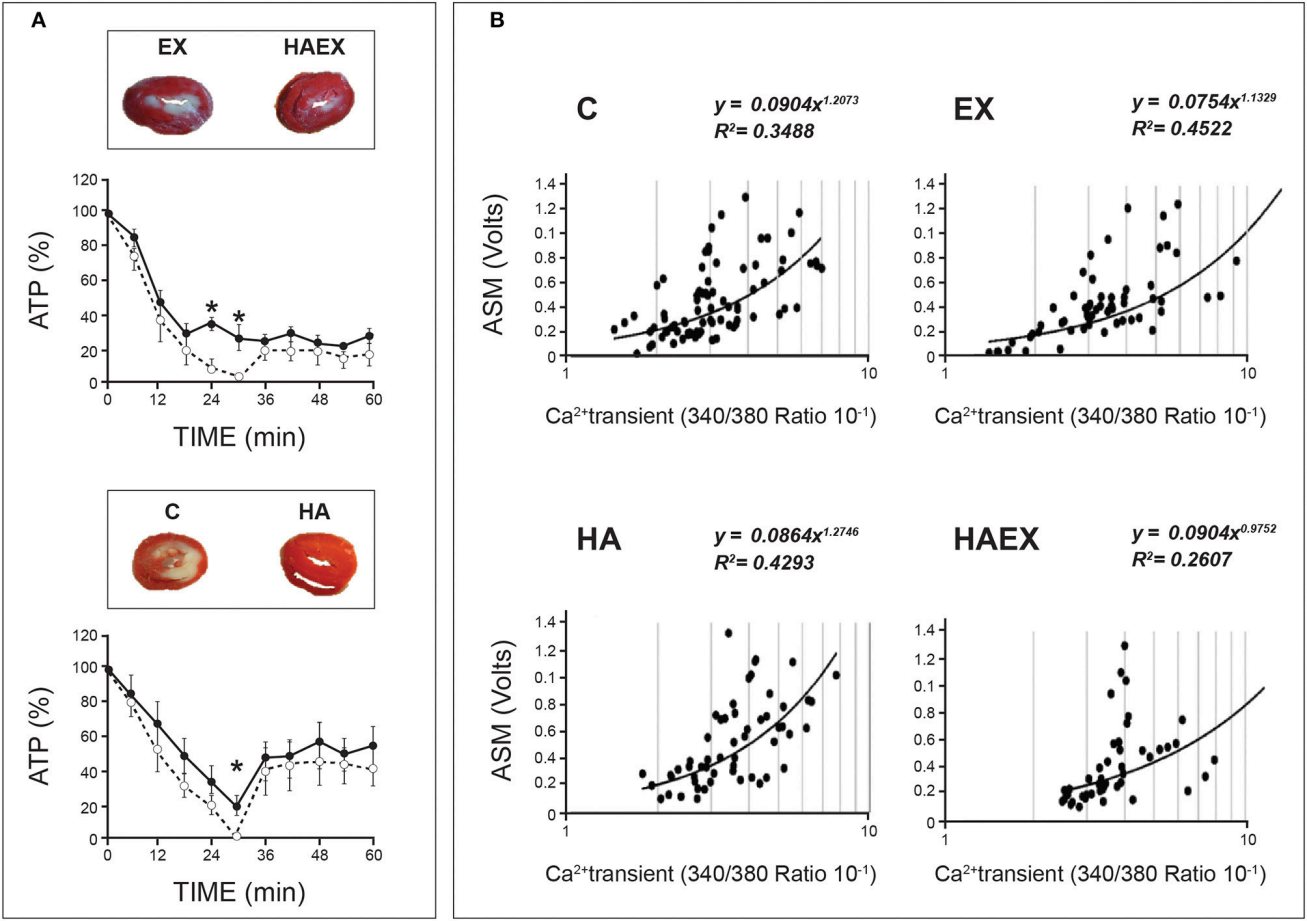
Demonstration of heat acclimatory features linked to HACT in rat hearts. Rats were acclimated either to passive heat acclimation (HA) or to HA and swimming training (HAEX). **(A)** Phospho-NMR monitoring of %ATP in rat hearts subjected to global ischemia/reperfusion (I/R) insults. Groups exposed to heat maintained significant ATP levels following 30 min of global ischemia (vs. non-acclimated rats' heart). Solid lines: Heat-exposed groups (HA & HAEX). Broken lines: Non-acclimated controls (C & EX). Insets: Heart slices post 30 min ischemia/40 min reperfusion. **(B)** Ca^2+^ sensitivity of isolated cardiomyocytes expressed by contractile response (ASM), vs. calcium transients. Data from the two heat-exposed groups yielded a rightward shift of the curves, suggesting decreased Ca^2+^ sensitivity. HA, Heat acclimation. HAEX, Heat acclimation and swimming training. EX, Swimming training alone. C, Control (Non-acclimated). Adapted from (Levi et al., 1993; Levy et al., 1997; Kodesh et al., 2011). Courtesy of the American Physiological Soc.

A particularly interesting aspect of HACT lies in the outcome of concomitant exposure to a second adaptagent, with opposing demands, during HA. If HACT indeed occurs, it constitutes the outcome of additive or cross-over effects. For example, HA and exercise training (HAEX), are usually employed in studies of human HA. Aerobic exercise training boosts muscle performance; consequently, biochemical adaptations enhance aerobic and anaerobic energy metabolism to meet the need for greater ATP generation. These alterations seem to be in conflict with critical adaptive features of HA, such as a decreased basal metabolic rate. Proceeding with the example of HACT against ischemia, Figure 2 shows, in rat hearts, that despite adaptive conflicts between heat and exercise, similar cardioprotection, as determined by infarct size, was achieved in the HA alone, in HA combined with swimming training (HAEX), and in normothermic swimming-trained (EX) groups (Levi E MSc, The Hebrew University 2002 and (Levy et al., 1997). The heat-treated rats maintained higher ATP levels in their ischemic cardiac muscles than that of the non-acclimated animals (Levi et al., 1993; Levy et al., 1997; Figure 2A); however, we have no unequivocal experimental evidence that under ischemic conditions, the impact of HA is central to the cardioprotection seen in the acclimated/trained phenotype.

The HA phenotype (passive heat exposure as a singular adaptagent), however, displays a variety of specific features that can be cardioprotective, in and of themselves. Thus, a deeper understanding of the roles played by environmental heat vs. metabolic heat load (achieved by training) in the protected phenotype may enable us to determine the origins of the protective features in the aforementioned experimental groups. Here, we are referring to decreased Ca^2+^ sensitivity (and, in turn, protection from Ca^2+^ overload) seen in both the HA and HAEX, but not in the EX groups (Figure 2B; Cohen et al., 2007; Kodesh et al., 2011). These findings suggest that the consequences of prolonged heat exposure predominate in this case, at least (see ***Phenotypic plasticity underlying HACT—a broad perspective***, below, for further discussion).

In a review by Powers et al. (2014), the authors stated that in exercise training cross-tolerance, the mitochondrial phenotype is central to exercise-induced cardioprotection “through increased expression of beneficial antioxidant proteins and decreased expression of proteins with potentially deleterious functions”. Our bioinformatic data imply that equally enhanced redox buffer systems are implicated in adaptive responses in HA, EX, and HAEX hearts (Kodesh et al., 2011). Currently, however, data from our models are insufficient to attribute a central protective role to these buffer systems.

Pollak et al. (1998) in a novel study of “translational medicine” on human patients, were the first to demonstrate that HACT, arising out of concomitant exposure to a second adaptagent with opposing demands during HA, is beneficial for humans with coronary artery disease. The impact of HA with modest exercise training on patients undergoing coronary artery bypass surgery was evaluated, using transesophageal echocardiography in conjunction with simultaneous hemodynamic monitoring immediately after the surgery. Findings demonstrated that HA protected chamber elasticity and improved diastolic function in the heat-acclimated patients, vs. increased post-surgery stiffness developed in the non-acclimated cohort (control).

### Cytoprotection-HIF-1α (hypoxia inducible transcription factor-1α)

Observations of the HA-protected rat cardiophenotype indicate: (i) enhanced glycolytic capacity, though at a slower rate; (ii) larger pre-ischemic endogenous glycogen stores; and (iii) upregulation of the transcript phosphofructokinase 2 (PFK2), a rate-limiting glycolytic enzyme in the normoxic HA rat heart (Eynan et al., 2002). The regulation of these components by HIF-1α (Semenza, 2011) led us to hypothesize that HIF-1α, the master regulator of oxygen homeostasis, is also an important mediator of HACT. This hypothesis was confirmed by Maloyan et al. (2005), and Shein et al. (2005), who were the first to show that HA induces constitutive upregulation of HIF-1α, independent of O_2_ levels in the rat heart and mouse brain subjected to cardiac ischemia and TBI, respectively.

HIF-1α exerts its transcriptional activation as the heterodimer HIF-1, the product of HIF-1α with the constitutive HIF-1β. Its role in cardio-HACT and neuro-HACT was confirmed by instituting a HIF-1α dimerization blockade either prior to ischemia in the heart, or prior to recovery from traumatic brain injury. In the rat, a HIF-1α dimerization blockade was also conducted during the entire 1 month HA session. We have shown that a continuous HIF-1α dimerization blockade is needed to attenuate HACT, even though no changes in acclimatory levels of HSP72 were measured (Alexander-Shani et al., 2017). his attenuation adds metabolic aspects to HACT. In the HA hearts, inhibition of HSP72 induction by β adrenergic blockade during acclimation, abolished HACT (Maloyan and Horowitz, 2002). A β adrenergic blockade was also shown to decrease HIF-1α induction (Li et al., 2015). In retrospect, the experimental series in which the magnitude of HACT was measured (e.g., infarct size) during blockade of HIF-1α dimerization and HSP72 augmentation, serve as examples of the integrated beneficial effects needed to achieve HACT. More recently, experiments involving HIF-1α mitochondrial target genes demonstrated the positive contribution of HIF-1α to HACT by shifting pyruvic acid away from the mitochondria to enhance glycolysis (Alexander-Shani et al., 2017). The beneficial effects of HA in experimental animal models will be further discussed below (see “*Phenotypic plasticity underlying HACT—a broad perspective”*).

## Cellular cytoprotection and HACT—confirmation of the role played by HSP and HIF-1α in humans undergoing heat exercise and hypoxia acclimation

In humans, Yamada et al. demonstrated HA-induced expression of HSP70 during exercise (Yamada et al., 2007). Consequently, by using the *hsp70* transcript as an indicator of strain, Lee et al. (2016) and Gibson et al. (2015) demonstrated HACT to hypoxia, also in humans. Notably, the acclimation protocols used by Gibson et al. (2015); Lee et al. (2016) included both heat and exercise, thereby examining the contribution of HA to HACT with co-adaptagents with conflicting/competitive demands, when adjusting to environmental demands. Supporting evidence of the diverse roles played by HIF-1α in HACT is derived from Lee et al. (2016), who compared the impact of HA and exercise to that of hypoxia acclimation and exercise, and demonstrated that HA elevates extracellular levels of HIF-1α, thus confirming in humans that HA induces HIF-1α under normoxic conditions. Likewise, the authors demonstrated that HA improves performance under hypoxic conditions, and that HA increases cellular and systemic physiological tolerance to exercise under conditions of moderate hypoxia. Moreover, the authors showed that HA improves performance during hypoxia in a manner similar to that achieved by hypoxic acclimation. As only extracellular HIF-1α was measured, the specific role of HIF-1α in HACT cannot be assessed.

## HACT: abrupt post-insult release of help signals

Unfortunately, the role of abrupt post-insult release of help signals has been less studied; hence, it will only be briefly discussed here. Empirical data were collected from ischemic heart and mouse brain models. In the heart, a noteworthy finding that emerged was the earlier activation threshold (upregulation) of *hsp72* and the glutathione-*S*-transferase P subunit (*GST-P*-antioxidant) to ischemic insult, and the downregulation of the *bcl-2* death promotor (BAD) to heat stress, after heat acclimation (Horowitz et al., 2004). Of prime interest was the finding of enhanced help signals evoked following traumatic head injury in the mouse brain, demonstrating angiotensin-2 (AT2) receptor induced neurogenesis, and acute activation of the AKT-HIF-1a cascade (Akt, HIF-1, GLUT1, VEGF, NGF, BDNF, and Erk1/2). For further information, readers are referred to Umschweif et al. (2014).

## Phenotypic plasticity underlying HACT: a broad perspective

Unequivocal evidence underscores the important role played by HSP72 and HIF-1α in HACT against impairment in oxygen homeostasis, possibly only because of elevated constitutive levels, which mitigate the need for immediate *de novo* induction upon stress. However, HA also exerts a global protective effect that is implemented in the featured HACT. Here, the impact of (i) the HIF-1α metabolic switch, and (ii) how HA induces processes to cope with the deleterious effects of Ca^2+^, both studied extensively in our laboratory, will be discussed as prototypes.

HIF-1α is an O_2_-regulated subunit; however, HA upregulates HIF-1α under normoxic conditions. An important adaptive strategy of the HA phenotype to decreased oxygen availability stems from its reliance upon enhanced glycolysis at a slower rate (vs. normothermic animals; Eynan et al., 2002), balancing pH with greater cytosolic ATP production and decreased mitochondrial ROS production. The engine that sets this adaptation in motion is the HIF-1α-mediated elevation of pyruvate dehydrogenase kinase 1 (PDK1) and, in turn, pyruvate dehydrogenase (PDH) activity, shifting pyruvic acid away from the electron transport machinery (Alexander-Shani et al., 2017). Activation of glycolytic enzymes is thus enhanced, while mitochondrial ROS production is diminished, due to decreased aerobic respiration. Canaana (2003) and Horowitz et al. (2006) demonstrated this outcome in HA rat cardiomyocytes, while measuring mitochondrial ROS production during heat stress and anoxia in the presence and absence of complex I-III inhibitors. Furthermore, the performance of the respiratory complex I, II and IV of HA mitochondria following ischemia/reperfusion insult was enhanced, compared to non-acclimated mitochondria (Assayag et al., 2010). In tandem with this important metabolic adaptive response is the HIF-1α-controlled reprogramming of two COX4 isoforms (components of Complex IV), elevating levels of the COX4.2 hypoxic-resistant unit (Alexander-Shani et al., 2017). Notably, the metabolic shift described above was first identified as altitude-induced adaptation in humans (Papandreou et al., 2006).

Experimental data suggests that remodeling of the COX4 isoform ratio is only one example of the principle involving remodeling of the subunit ratio of protein complexes, leading to functional changes and performance as protective mechanisms in HACT, conditioned to “attenuate injury”. Both the heart and brain use this principle to alter Ca^2+^ management, and to reduce the deleterious effects of Ca^2+^ overload. In rats, the cardiac heat-acclimated phenotype demonstrates reduced Ca^2+^ sensitivity; namely, more Ca^2+^ is needed to induce cardiac muscle contraction (Cohen et al., 2007; Kodesh et al., 2011). In cardiac mitochondria, serving also as a Ca^2+^ sink, Ca^2+^ levels increase in these organelles without affecting their performance (Assayag et al., 2012). The frontal cortex and hippocampus display decreased normoxic NMDA density, with an even greater decrease noted during hypoxic stress. Upon exposure to hypoxic/ischemic stress, a massive glutamate discharge is seen, causing calcium overload and oxidative stress. The combination of fewer NMDA receptors in the acclimated phenotype, coupled with an increase in the NMDA receptor subunit ratio favoring decreased Ca^2+^ permeability, is likely to reduce Ca^2+^ overload, and attenuate its deleterious effects in the stressed brain (Yacobi et al., 2014).

**Figure 3:**
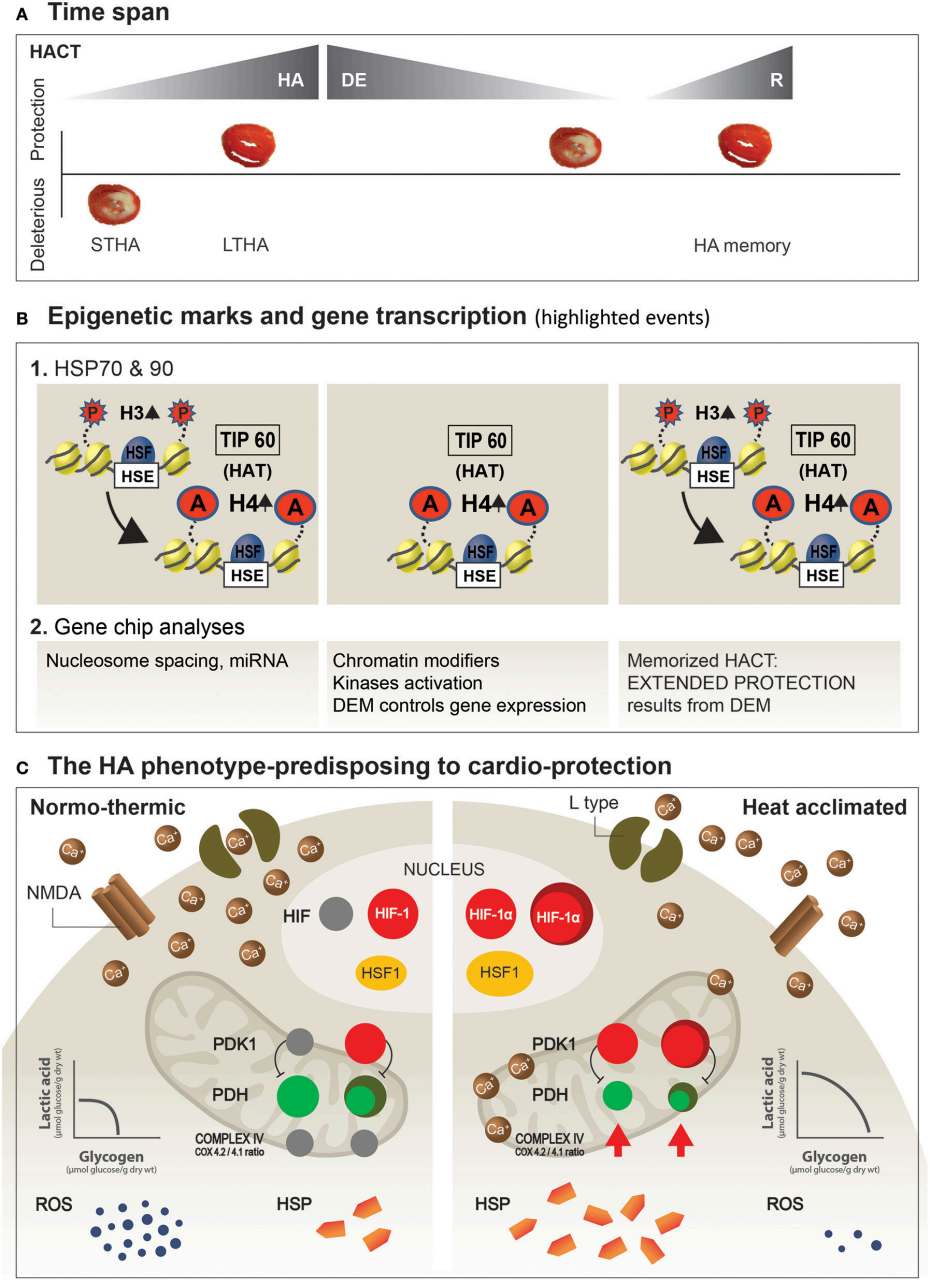
Suggested dynamics of epigenetic machinery and highlighted mechanisms shaping the HA-protected phenotype. **(A)** HA and HA memory time span. Ischemic/reperfused heart slices illustrate protective and deleterious responses. **(B)** Post-translational modifications in histones (histone H3-P; histone H4-Act) underlie heat acclimation-mediated cytoprotective memory. The onset of acclimation triggers histone H3-P, tagging on LTHA, to H4 acetylation at the HSP promotor (Cheung et al., 2000). Long-term histone H4 acetylation promotes a constitutive open chromatin at the HSP promotor and constitutive HSF1 binding long after the initial HA, as well as during re-HA (For a detailed explanation, see in “Future perspectives” above). **(C)** Protective features developed in the HA phenotype.The illustrated features are those described in the main text. Left side: Normothermic heart. Right side: HA heart. Increased expression of HSF1 leads to augmented constitutive cellular HSP levels, while constitutive HIF-1α marks the metabolic switch, which upregulates mitochondrial PDK1. In turn, phosphorylation of ser(232) in the PDHE1α subunit of the PDH complex shifts pyruvic acid away from the mitochondria, to enhance glycolysis and to decrease ROS produced by the mitochondria. An additional, important aspect of HA is the “principle” of receptor remodeling, or abundance of Receptors/ion channels on the cell membrane (i.e., heart, brain) as a mechanism to attenuate the deleterious effects of Ca^2+^ overload. STHA, Short-term heat acclimation; LTHA, Long-term heat acclimation; DEM, Dynamic epigenetic machinery (Based on Eynan et al., 2002; Horowitz et al., 2004; Tetievsky and Horowitz, 2010; Yacobi et al., 2014; Alexander-Shani et al., 2017).

The unifying principle underlying changes in Ca^2+^ management in these two organs entails remodeling of the Ca^2+^ channels. In the heart, the gating subunit of the L-type channel (Kodesh et al., 2011), and in the NMDA (Yacobi et al., 2014), an elevation of the GluN2B/GluN2A NMDA receptor subunit ratio (to ratios >1), together with a significant increase in the GluA2 subunit of the AMPA receptors, lead to decreased opening permeability of the channels which, in turn, limits Ca^2+^ flux during stress. For a scheme describing highlighted changes leading to HACT, see Figure 3, lower panel.

## HACT: translational aspects to humans' health

The beneficial effects of heat acclimation have been used for human benefit long ago. But while HA protocols were initially developed to reduce the physiological strain caused by hot environments, the cellular and molecular processes underlying HA were consolidated only recently. Consolidating the cellular and molecular mechanisms of acclimation led to the emergence of recent investigations/publications demonstrating that HA can be exploited for human benefit beyond its evolutionary function of “heat tolerance”. In humans, Lee et al. (2016) demonstrated improvements in hypoxic exercise tolerance after 10 days of HA; Brunt et al. (2016a,b) showed decreased vascular stiffness following 8 weeks of prolonged thermal therapy. Interestingly, Pollak et al. (1998) reported decreased cardiac chamber stiffness immediately post-coronary bypass surgery, undertaken after 2–3 weeks of acclimation in early-summer desert temperatures. We can only now, provide partial explanation to these finding. The accumulated data raise questions regarding the time required for HACT. In rodents, for example, HACT is achieved following 3–4 weeks of HA. Are time differences in the induction of HACT the result of species variation? Considering HA in humans, it is important to bear in mind that for practical reasons, we examine physiological acclimation, rather than molecular processes. The physiological processes occur within a shorter timeframe than those required to achieve cellular and molecular homeostasis, and are controlled by dynamic neural processes. With the few available molecular cytoprotective responses (Gibson et al., 2015, 2016; Lee et al., 2016), and cellular processes (e.g., muscles' contractility, Racinais et al., 2017) it is likely that acclimatory features are similar in humans and rodents. Our data suggest that acclimation constitutes a gradually progressive process. It is likely that in the short-range human protocols (~2 weeks), full acclimatory homeostasis cannot be achieved. Molecular acclimatory homeostasis, on the other hand, entails epigenetic processes of gene transcription and may thereby enable extension of the therapeutic windows discussed below (“Future perspectives”).

## Future perspectives: do epigenetic mechanisms play a role?

A hallmark of HACT is the extended time for which the animal is protected: the effects of cross-tolerance last ~3 weeks post-acclimation. HACT is characterized by a unique ability to be memorized and reinstated, even after 1 or even 2 months of decline. A short acclimating stimulus of only 2 days (vs. 30 days for initial acclimatory homeostasis) was required for the return of HACT (Tetievsky et al., 2008).

Accordingly, epigenetics attracted our attention. Using *hsp72* and *hsp90* in a rat heart model for studying post-translational histone modifications (H3 phosphorylation, H4 acetylation), Tetievsky et al. (Tetievsky and Horowitz, 2010) demonstrated that the memorized HACT stems from within-life epigenetic mechanisms controlling gene expression.

Moreover, bioinformatic analyses of the acclimated, de-acclimated, and re-acclimated transcriptomes provided evidence that epigenetic processes exist on a continuum, occurring during acclimation and de-acclimation, that are likely to enhance both the protected HA phenotype, and the rapid re-induction of HACT. Among the processes involved are: (i) changes in the linker histones participating in nucleosome spacing, transcription factor accessibility and, in turn, gene expression control during (ii) de-acclimation, a molecular set-up whereby constitutive histone H4 acetylation by the histone-acetylase Tip60 enables HSF1 (heat shock transcription factor 1) binding to the heat shock element, thus predisposing to maintain HSP72 proteostasis, and a return of HACT (Tetievsky et al., 2008, 2014; Tetievsky and Horowitz, 2010). Notably, there exists a dichotomy between the transcriptome of the reinstated, protected, and HA phenotypes, while the physiological phenotype is similar in both phases. Central signaling pathways are most active during the transition from normothermic to acclimated temperatures, perhaps implying that ambient temperature has an impact on HACT.

Corroborating our postulation of the importance of epigenetics in cross-tolerance is the review by Khoury et al. (2016) on the recapitulation of evolutionary metabolic plasticity by means of epigenetic mechanisms. Pharmacological preconditioning by administering resveratrol, an activator of the epigenetic enzyme Sirtuin1 (SIRT1) resulted in a cross-tolerance lasting ~14 days, by “mimicking” the depressed metabolic state and enhanced hypoxic tolerance, an evolutionary adaptive feature among hypoxic-tolerant species (Storey, 2015). Thompson et al. (2013) demonstrated bimodal activity of the histone deacetylase SIRT1 in ischemic brain protection, seen as either hypermethylation and gene suppression, or hypomethylation and gene activation.

Gidday (2015), in a review published in Frontiers in Neurology (hypotheses and theory articles) entitled, “Extending injury- and disease-resistant CNS phenotypes by repetitive epigenetic conditioning,” discusses the idea of broadening ischemic tolerance in the brain by “extension of the period over which adaptive epigenetic changes persist, as well as its memory” and suggests that “it is epigenetics that deserves attention as the fundamental mechanism responsible for the long-lasting responses to repetitive conditioning stimuli”. Horowitz's lab (Tetievsky et al., 2008, 2014; Tetievsky and Horowitz, 2010) provided experimental physiological and epigenetic evidence in a rat heart that a within-life epigenetic mechanism induces memorized HACT. Empirical evidence points toward constitutive histone H4 acetylation on the HSP72 promotor, and upregulation of this cytoprotective transcript following long-term acclimation when HA homeostasis has been achieved. Histone H4 acetylation is absent following short periods of acclimation (Tetievsky and Horowitz, 2010). However, histone H4 acetylation is maintained throughout acclimation, acclimation decline, and re-acclimation, suggesting an “epigenetics” imprinting of the long-, but not short-term stressful episode (Figure 3B).

In sum, the dynamic epigenetic phenomenon not only induces long-lasting HACT, but enables preservation of its physiological beneficial features in a dormant manner. A rapid, short acclimation stimulus re-establishes the physiological, protected HA phenotype. The transcriptional machinery, however, is a continuum. HACT has beneficial roles in health and disease and is already employed in thermotherapy. Yet, our knowledge of the epigenetic within-life dynamic mechanisms involved, as well as how long they persist, is still in its infancy.

## Author contributions

The author confirms being the sole contributor of this work and approved it for publication.

### Conflict of interest statement

The author declares that the research was conducted in the absence of any commercial or financial relationships that could be construed as a potential conflict of interest.

## Abbreviations

HA: heat acclimation
HACT: heat acclimation mediated cross-tolerance
HAEX: heat acclimation combined with exercise training
EX: exercise training
COX: cytochrome c oxidase
HIF-1α: hypoxia inducible transcription factor 1α
HSP: heat shock protein (number denotes molecular weight)
NMDA: N-methyl-D-aspartate
ROS: reactive oxygen species.
